# Fetal Heart Rate Analysis for Automatic Detection of Perinatal Hypoxia Using Normalized Compression Distance and Machine Learning

**DOI:** 10.3389/fphys.2017.00113

**Published:** 2017-02-28

**Authors:** Óscar Barquero-Pérez, Ricardo Santiago-Mozos, José M. Lillo-Castellano, Beatriz García-Viruete, Rebeca Goya-Esteban, Antonio J. Caamaño, José L. Rojo-Álvarez, Carlos Martín-Caballero

**Affiliations:** ^1^Department of Signal Theory and Communications, University Rey Juan CarlosFuenlabrada, Spain; ^2^Department of Obstetrics and Gynaecology, Hospital Universitario Fundación de AlcorcónMadrid, Spain

**Keywords:** fetal heart rate, perinatal hypoxia, normalized compression distance, heart rate variability, information theory

## Abstract

Accurate identification of Perinatal Hypoxia from visual inspection of Fetal Heart Rate (FHR) has been shown to have limitations. An automated signal processing method for this purpose needs to deal with time series of different lengths, recording interruptions, and poor quality signal conditions. We propose a new method, robust to those issues, for automated detection of perinatal hypoxia by analyzing the FHR during labor. Our system consists of several stages: (a) time series segmentation; (b) feature extraction from FHR signals, including raw time series, moments, and usual heart rate variability indices; (c) similarity calculation with Normalized Compression Distance, which is the key element for dealing with FHR time series; and (d) a simple classification algorithm for providing the hypoxia detection. We analyzed the proposed system using a database with 32 fetal records (15 controls). Time and frequency domain and moment features had similar performance identifying fetuses with hypoxia. The final system, using the third central moment of the FHR, yielded 92% sensitivity and 85% specificity at 3 h before delivery. Best predictions were obtained in time intervals more distant from delivery, i.e., 4–3 h and 3–2 h.

## 1. Introduction

Perinatal hypoxia is a fetus and newborn child disease resulting from the lack of tissues oxygenation. Although it can occur in earlier gestation phases, childbirth and immediate neonatal hours are the fundamental risk periods. The perinatal hypoxia severity spectrum ranges from very mild cases (only requiring neonatal resuscitation with environmental oxygen), more serious cases requiring intubation and acidosis correction with bicarbonate (reanimation types V and VI) and critical cases that can cause perinatal death or serious damage, such as brain or adrenal hemorrhage, necrotizing enterocolitis, delayed neurological development, mental disability, seizures (West syndrome) or cerebral palsy (Leuthner and Das, 2004; Morales et al., 2011). Diagnosis is performed at the time of birth by evaluating the cardio-respiratory depression and muscle tone. The severity of the hypoxia is commonly quantified using the Apgar Score (Apgar, 1953; Casey et al., 2001), with a score lower than 7 at 5 min after delivery being considered as pathological, which is usually confirmed with gas analysis of the umbilical cord.

Continuous electronic fetal monitoring, also known as Cardiotocography (CTG), was developed around 1960 (Hon, 1958; Hammacher et al., 1968) and consists in the simultaneous evaluation of the Fetal Heart Rate (FHR) and the uterine activity. After CTG generalization, two relevant signs of suspicious fetal hypoxia were recognized, namely, the late deceleration of the FHR in relation to uterine contractions, and the FHR variability decrease (Low et al., 1999). Although visual interpretation of CTG has an acceptable sensitivity for risk of hypoxia detection (especially in pathological traces), the specificity is still low (especially for suspicious traces), and requires the confirmation with invasive pH determination of the fetus' scalp blood, which is technically cumbersome and not always feasible (Tasnim et al., 2009). When considering the risk of hypoxia, gynecologists prescribe intervention (cesarean, forceps, and vacuum extraction) more often than necessary (Tasnim et al., 2009), thus increasing sensitivity at the expense of specificity. In addition, visual assessment of bradycardias and late deceleration is simple, whereas visual assessment of the loss of variability is not and even varies depending on the observer, representation type (computer display or paper), or cardiotocograph model (Bernardes et al., 1997; Ayres-de Campos et al., 1999; Santo and Ayres-de Campos, 2012).

Automated signal processing methods for supporting the gynecologists in the early hypoxia detection need to be working on hard to process time series, with different time durations, recording interruptions for seconds or even minutes, and poor quality signal conditions. Aiming to overcome these limitations, we present a method for automated detection of perinatal hypoxia from FHR time series registered during labor, which is specifically designed to be robust in those conditions. Our method consists of several stages: (a) a first stage for time series segmentation; (b) followed by the design and analysis of a feature extraction subsystem, based on the use of the raw FHR time series, its statistical moments, and usual heart rate variability (HRV) indices; (c) the Normalized Compression Distance (NCD), (Li et al., 2004), which is closely related to the Kolmogorov Complexity and mutual information (Cover and Thomas, 2006), is subsequently used for dissimilarity estimations between time series with different lengths and recording interruptions; (d) finally, a classification algorithm is used to provide the estimated hypoxia detection output. The method design alternatives have been benchmarked on a database with 32 fetal recordings (15 controls).

The structure of the paper is as follows. Section 2 describes the alternative elements of the detection system. Section 3 describes the FHR dataset and Section 4 then experimentally demonstrates the capability of NCD both for classification of raw signals and for extending the capabilities of conventional analysis in a real FHR dataset. Finally, Section 5 discusses the main advantages of the proposed methodology over other alternatives and presents our conclusions.

## 2. Methods

The proposed system consists of several stages, namely: time series segmentation and feature extraction from FHR signals; similarity calculation with NCD; and the choice of a suitable classification algorithm for the final purpose of hypoxia detection. The theoretical basis and design criteria for these stages are described below.

### 2.1. Time series segmentation and feature extraction

#### 2.1.1. Time series segmentation

We decided to analyze the FHR signal in 1-h windows, in order to determine the accuracy that can be attained at 3, 2, and 1 h intervals before delivery. This will show whether hypoxia signs can be detected at such time milestones, thus allowing decisions to be made as quickly as possible on stressed fetuses. We also considered the signal segment from 4 to 1 h before delivery, with the aim of simulating a real situation in which the remaining labor time is unkown. Finally, we also analyzed the FHR signals by dividing them into a set of short sliding windows (5 min segments), which is common practice in heart rate signals analysis (Signorini et al., 2003).

#### 2.1.2. Feature extraction

Feature extraction techniques aim to gather specific parameters from a signal that can be easier to analyze than the signal samples themselves. The use of raw data is theoretically supported by data processing inequality,according to which, signal processing cannot increase the information content (Cover and Thomas, 2006). However, information loss caused by feature extraction is preferable to raw data analysis, as it simplifies subsequent classification or estimation. In this work, we consider several types of features. Firstly, the HRV parameters that are commonly used for analyzing heart signals in different applications (Task Force, 1996), and they require their own preprocessing stage. Secondly, statistical moments that can be considered as generally used parameters for characterizing signals in general. Although statistical moments discard the temporal structure of a time signal, they are known to be robust to signal loss and easy to compute.

#### 2.1.3. FHR features from HRV conventional analysis

FHR conventional analysis is often performed using time domain and frequency domain indices computed in 5 min segments. For linear HRV analysis, a preprocessing algorithm is applied to the raw signal to deal with noise and artifacts related to the fetal and maternal movements. Beats lower than 60 beats per minute (bpm) and beat-to-beat differences higher than 25 bpm are identified by the preprocessing algorithm as noise or artifacts. Beats labeled by the acquisition machine as *lost* (see Section 3) are also identified as artifacts (Signorini et al., 2003; Gonçalves et al., 2006). Every beat labeled as an artifact is then removed and replaced using linear interpolation. Segments with more than five consecutive beats identified as artifacts or with more than 5% of artifacts are discarded for the analysis. FHR recordings are exported from commercial cardiotocographs as a digital signal sampled at 4 Hz, so FHR signals are subsequently downsampled from 4 to 2 Hz (following Gonçalves et al., 2006), keeping only the odd samples.

Let *s*[*n*], for *n* = 1, …, *N*, be the set of values of FHR signal, also denoted by **s** in vector form. The following time domain indices (Magenes et al., 2000) can be computed:

(1) $$\begin{array}{l} \bar{FHR}=\overset{-}{s}=\frac{1}{N}\overset{N}{\underset{n=1}{\sum }}s\left[n\right] \end{array}$$

(2) $$\begin{array}{l} stdFHR=\sqrt{\frac{1}{N-1}\overset{N}{\underset{n=1}{\sum }}{\left(s\left[n\right]-\overset{-}{s}\right)}^{2}} \end{array}$$

(3) $$\begin{array}{l} LTI=\text{IQR}\left(\left\{\sqrt{s^{2}\left[n\right]+s^{2}\left[n+1\right]},\;1\leq n\leq N-1\right\}\right) \end{array}$$

(4) $$\begin{array}{l} STV=\frac{1}{24M}\overset{24M}{\underset{n=1}{\sum }}|sm\left[n+1\right]-sm\left[n\right]| \end{array}$$

where IQR denotes the inter-quartile range, *M* the number of the minutes in the segment under analysis, *sm*[*n*] the value of the signal *s*[*n*] taken each 2.5 s (i.e., once every five samples) and *std* means standard deviation.

Frequency domain indices are computed by using nonparametric spectral estimation based on the Welch periodogram, with a *Hanning* window, on 256 samples segments, and with 50% overlapping (Bernardes et al., 2008). The mean and linear trend are subtracted before calculating the periodogram. Frequency domain indices to assess FHR variability are computing as the total power in different frequency bands, which are (Signorini et al., 2003): Very Low Frequency, *P*_*VLF*_, in the band (0, 0.03) *Hz*; Low Frequency, *P*_*LF*_, in the (0.03, 0.15) *Hz*; Movement Frequency, *P*_*MF*_, in the band (0.15, 0.5) *Hz*; and High Frequency, *P*_*HF*_, in the band (0.5, 1) *Hz*. LF and HF bands defined here are associated with the fetal autonomic nervous system (ANS) regulation (Task Force, 1996). MF band, which corresponds to the HF band of classical HRV analysis, is related to fetal movements and maternal breathing of FHR signals (Gonçalves et al., 2006). Total power (*P*_*T*_) and the ratio $P_{LF}/\left(P_{MF}+P_{HF}\right)$, which quantify the balance of the ANS, are also computed as frequency domain indices.

#### 2.1.4. Features from statistical moments

Moments are simple descriptors of the shape of the distribution of a random set of values (Fisher and Cornish, 1937). They have been useful in many signal processing problems (Soliman and Hsue, 1992; Shi, 2005), and they are robust to signal loss, as they can be computed on the known signal samples, while ignoring the unknown time periods. The *kth*-order raw moment and the corresponding central moments are defined as:

(5) $$M_{k}(s)=\frac{1}{N}\overset{N}{\underset{n\;=\;1}{\sum \;}}s{\left[n\right]}^{k}$$

(6) $$\mu_{k}(s)=\frac{1}{N}\sum_{n\;=1}^{N}(s[n]-M_{1}(s){)}^{k}.$$

#### 2.1.5. Feature selection

Feature selection techniques search for the variable subset that provides the maximum information, while trying to avoid redundancy amongst them (Guyon and Elisseeff, 2003). This provides three benefits for the resulting models: improved generalization, better interpretability and shorter training and execution times. Among these techniques, wrapper methods build a model for each candidate set and select the model with the best performance in a validation set. Forward selection (FS) iteratively adds the non-included feature providing with the best accuracy in the training set to the included feature subset. The number of features is automatically selected in the training set as the minimum number that reaches maximum accuracy. In order to control overfitting, each candidate feature set can be evaluated by 2-fold cross-validation of the training sample.

### 2.2. NCD and similarity in time signals

A simple approach to classification is to assign to the test object the label of the closest or most similar object in a training dataset. The accuracy of this approach depends on the goodness of the distance measure for representing differences and similarities between the objects to be classified. The best measure would match all the common patterns between the objects, at the same time that it detects their differences. With a given object (in our case, signal **s**), similarity learning (Pekalska and Duin, 2002) uses as features the similarities ({*d*(**s**,**t**_*i*_)} for *i* = 1, …, *N*_*T*_) to a labeled training set {*y*_*i*_} of *N*_*T*_ objects. A machine learning classifier can then be readily trained by using these features. Indeed, the classifier is trained assuming that each instace *bx*_*i*_ is the *i*-th row of the square *N*_*T*_ × *N*_*T*_ matrix of the similarities *d*(*t*_*i*_, *t*_*j*_).

We choose a general similarity measure that is based on the common information among the signals and which can handle both linear and nonlinear relations between them. The Kolmogorov Complexity *K*(**s**) of a signal **s** is the length of the shortest binary program that produces **s** on an universal Turing machine (Kolmogorov, 1965; Li et al., 2004). Note that *K*(**s**) can be seen as the signal information (or the information required to generate it); *K*(**s**|**t**) is the length of the shortest program to produce **s** if **t** is given as an input; and *K*(**s**, **t**) is the length of the shortest program that generates **s**, **t**, i.e., the concatenation of **s** and **t**, and allows them to be separated. Up to an additive constant independent of **s**, and **t**, it can be proven (Li et al., 2004) that

(7) $$\begin{array}{l} K\left(t,s\right)=K\left(t\right)+K\left(s|t\right)=K\left(s\right)+K\left(t|s\right). \end{array}$$

The information distance between two signals is a similarity measure (Bennett et al., 1998) that can be defined as

(8) $$\begin{array}{l} ID\left(t,s\right)=\max\left\{K\left(s|t\right),K\left(t|s\right)\right\}, \end{array}$$

There are problems for practical use, namely, the Kolmogorov Complexity is not computable and we need a distance suitable for comparing signals of different sizes.

NCD is a similarity measure for signals (Li et al., 2004; Cilibrasi and Vitanyi, 2005). For two given signals **s**_*i*_, **s**_*j*_, the NCD(**s**_*i*_, **s**_*j*_) is defined as

(9) $$\text{NCD}(s_{i},s_{j})=\frac{C(s_{i},s_{j})-\min\{C(s_{i}),C(s_{j})\}}{\max\{C(s_{i}),C(s_{j})\}}\;,$$

where *C*(·) is the compression length in bits given by the selected compressor and *C*(**s**_*i*_) and *C*(**s**_*i*_, **s**_*j*_) the number of bits needed to compress **s**_*i*_ and the concatenation of **s**_*i*_ and **s**_*j*_, respectively. Note that *C* provides a computable approximation to the Kolmogorov Complexity. Three compressor types, namely zip, bzip2, and lzma, were compared in this work. This normalized measure is easy to interpret, in the sense that the lower its value, the more similar the signals. In other words, they share more information and fewer bits are required to compress both signals together. The normalization term in the denominator of Equation (9) enables the comparison of signals of different sizes. Also note that NCD values range from zero to slightly above one.

To the extent that NCD is only an approximation to the Kolmogorov Complexity, its performance can be improved by simplifying the compressor work. In other words, we can apply NCD to series of features, instead of applying it to the raw signals, with the aim of extracting the patterns that NCD is not able to resolve in the raw signals.

Similarity learning using NCD can handle more than one sequence type. For example, if we want to build a classifier with *J* series of time and frequency indices we have several alternatives:

- Concatenate all the series and proceed as in the case of only one series.
- Use one classifier per serie and vote for a predicted label.
- Combine each series similarity matrices into one, for example just by adding them, which can be interpreted as a soft version of the previous approach.
- Concatenate the similarity Matrices for each index to form an *N*_*T*_ × *J* · *N*_*T*_ instance matrix.

### 2.3. Classification engine

#### 2.3.1. Classification algorithms

On the one hand, the detailed physical model that generates the FHR records is complex and mostly unknown. On the other hand, we have some sets of available observations,however not enough data to estimate the conditional densities of the classes for diagnosis. We therefore propose using a non-parametric machine learning approach for classification and accordingly we take two approaches, namely, *k* Nearest Neighbors (*k*-NN), which is easy to combine with similarity measures, and Support Vector Machines (SVM), a state-of-the-art and advantaged classifier in a number of applications.

In a binary classification problem, we are given a collection of labeled samples {**x**_*i*_, *y*_*i*_} *i* = 1, …, *N*_*T*_, where $x_{i}\in {\mathbb{R}}^{D}$ and *y*_*i*_ ∈ {−1, 1}. The *k*-NN algorithm (Duda et al., 2000) selects the label for one test sample as the mode of the labels of the *k* training instances that are nearest to it (its *k* nearest neighbors). In the case of a tie, the decision can be taken at random or with the label of the closest neighbor. The distance between samples is defined by a similarity measure, which is usually the Euclidean distance, however in our case, it will be given by NCD instead. The asymptotic error of this simple classifier is bounded by twice the Bayes error, which is the minimum attainable error (Cover and Hart, 1967). In general, NCD similarity is not symmetric, and NCD(**s**_*i*_, **s**_*j*_) ≠ NCD(**s**_*j*_, **s**_*i*_). Therefore, to obtain the similarity between **s**_*i*_ and **s**_*j*_, we studied two types of similarity: type *min* using the minimum similarity, min{NCD(**s**_*i*_, **s**_*j*_), NCD(**s**_*j*_, **s**_*i*_)}, and type *mean* using the mean, 0.5(NCD(**s**_*i*_, **s**_*j*_) + NCD(**s**_*j*_, **s**_*i*_)).

SVM are powerful learning machines that can be easily trained and have been successfully used in many applications (Cortes and Vapnik, 1995; Schölkopf and Smola, 2001). The trained classifier for binary classification is the solution of the following convex optimization problem:

(10) $$\underset{{}_{w,b,\xi_{i}}}{\min}\frac{1}{2}||w|{|}^{2}+C\sum_{i}\xi_{i}$$

subject to:

(11) $$y_{i}(w^{⊤}\phi (x_{i})+b)\geq 1-\xi_{i}$$

(12) $$\begin{array}{r} \xi_{i}\geq 0 \end{array}$$

where **w** is the classifier solution and can be written as a combination of the training samples, i.e., $w=\sum_{i}\beta_{i}\phi (x_{i}).$ The objective function has two terms, the former a regularization term that penalizes rough solutions and the latter a term that penalizes classification errors, both being balanced by parameter *C*. Positive slack variable ξ_*i*_ accounts for the margin error of sample *i*, which enables solutions in non-separable problems; (·)^⊤^ is the transpose operator; ϕ() is a function projecting **x**_*i*_ into a possibly higher dimensional space where the linear classification is completed, which allows for non-linear classification functions in the original space ℝ^*D*^; and *b* is a bias term. The prediction for a new sample x^*^ is $y^{*}=\text{sign}(w^{⊤}\phi (x^{*})+b)=\text{sign}(\sum_{i}k(x^{*},x_{i})+b),$ where *k*(**x**_*i*_, **x**_*j*_) is a kernel that computes $\phi {\left(x_{i}\right)}^{⊤}\phi (x_{j})$ without explicitly evaluating ϕ(·). Here, we consider two kernel functions, namely, the linear kernel $k(x_{i},x_{j})=x_{i}^{⊤}x_{j},$ and the radial basis function kernel $k(x_{i},x_{j})=\exp\left(-\frac{||x_{i}-x_{j}|{|}^{2}}{2\sigma^{2}}\right),$ where σ defines the kernel width. Finally, Equation (11) shows that SVMs enforce a margin for classification, i.e., the label times the output of the classifier should be greater that 1, allowing for margin errors by incurring a penalty. Not all the margin errors are classification errors, but only those with ξ_*i*_ ≥ 1.

#### 2.3.2. Performance evaluation

In some applications where the labeled instances are scarce, a common approach is to estimate the performance of the classification in unseen test cases by cross-validation (Duda et al., 2000). In this paper, the accuracy of the different alternatives has been estimated by using leave-one-out (LOO) cross-validation. We choose the almost unbiased LOO accuracy estimation, even at the cost of its high variance, because we have a low number of examples in our dataset.

The complete procedure can be summarized as follows:

**for all** *i* **do**
      **X**_*T*_ = **X**\**x**_*i*_
      **Y**_*T*_ = **Y**\*y*_*i*_
      Feature selection: *FS* = *g*(**X**_*T*_, **Y**_*T*_)
      Classifier training: *f* = *h*(**X**_*T*_, **Y**_*T*_, *FS*)
      Prediction: $\hat{y}$_*i*_ = *f*(**x**_*i*_, *FS*)
**end for**
Performance evaluation: *p* = *L*(**Y**, $\hat{Y}$)

where $X=\{x_{i}\},Y=\{y_{i}\},\hat{Y}=\{{\hat{y}}_{i}\},\;i=1,\ldots ,N_{T}$; “\” means set subtraction (i.e., $X\backslash x_{i}=\{x_{1},x_{2},\ldots ,x_{i-1},x_{i+1},\ldots ,x_{N_{T}}\}$), *g, h* are feature selection and classifier training algorithms, respectively; *FS* are the selected features; and *L* is the 0–1 loss function.

**Figure 1:**
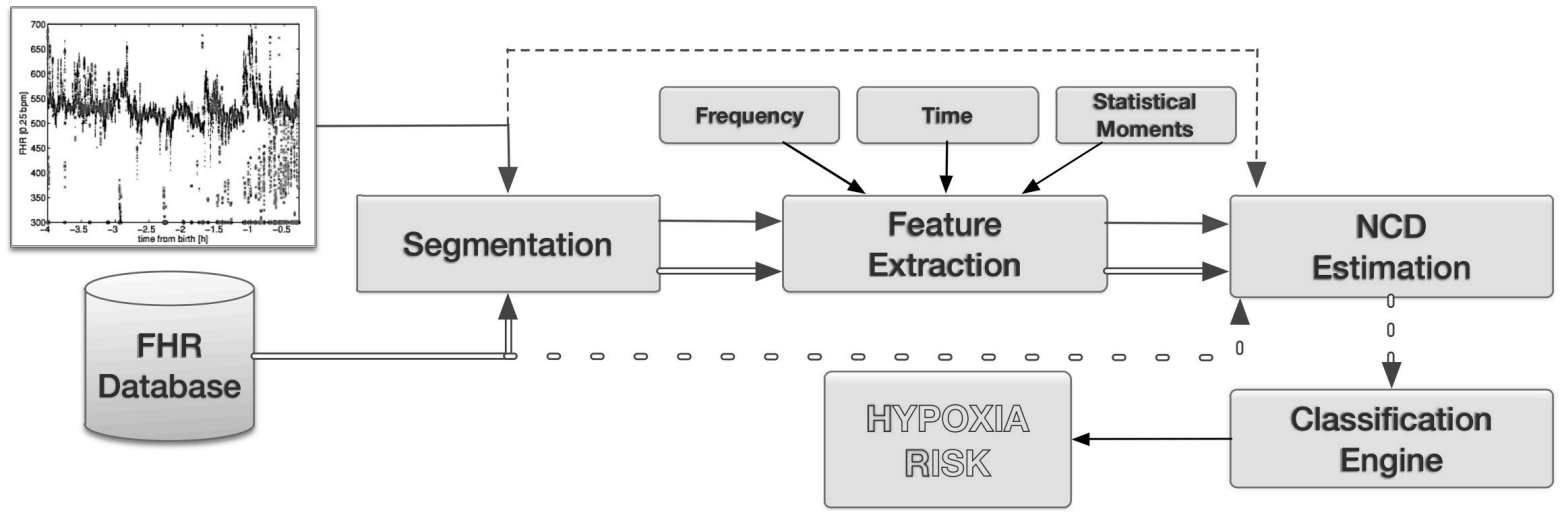
**Final system proposed in the paper**. A target FHR time series is, first, segmented; then features are extracted; and finally compared with a database of FHR using NCD. Feature selection could be performed after NCD stage. Optionally, raw FHR signals can be the input of the NCD stage without preprocessing (dotted lines). The output of the NCD are feature inputs for the classifier engine, which in turn produces a hypoxia risk.

Figure 1 shows a complete final schema of the proposed system. A new FHR recording to be evaluated is first segmented; then characteristic features are extracted, the raw FHR recording is a possible option indicated as an arrow from raw recordings to NCD stage directly; and finally, NDC is used to the recording to be evaluated compare with a gold standard database of FHR. Note that FHR from the database should pass trough the same preprocessing stages. Dissimilarity matrix, which is the output of the NCD stage, represents the input features for the classifier engine, which, in turn, prodives with an estimation of the hypoxia risk.

### 2.4. Experimental setup

In order to select the best combination of the elements in the system proposed in Figure 1, and to evaluate the performance of the final system, we propose the following experimental setup:

- We started by evaluating the classification performance in FHR raw signals, without any preprocessing, using only the NCD similarity criterion and a nearest neighbor classifier. This experiment evaluated the performance of NCD and set the baseline accuracy that could be attained (Section 4.1).
- We then considered as features for hypoxia classification the aforementioned time and frequency indices that are commonly used in HRV analysis, aiming to evaluate whether they showed any improvement over NCD raw analysis (Section 4.2.1).
- Subsequently, we analyzed the performance obtained by using as features the general purpose statistical moments applied to the raw signals, without using NCD (Section 4.2.2).
- We also performed feature selection on each group of variables (time and frequency HRV indices and statistical moments), in order to identify the best features and to see whether feature selection could improve classification performance (Section 4.2.3).
- The experiments continued by evaluating whether the NCD could empower the HRV parameters and statistical moments by obtaining at similarity of feature sequences (Sections 4.3.1 and 4.3.2). In these experiments, features were computed on sliding windows, taking into account the fact that physiological time series are not stationary.

The experiments were carried out in four time intervals, namely: the complete segment from 4 to 1 h before delivery, and for each single hour (4 ↔ 1,4 ↔ 3,3 ↔ 2, and 2 ↔ 1).

## 3. Data description

FHR records^1^ were acquired with a Philips cardiotocograph for a total of 32 recordings, 15 controls and 17 cases. A case was declared when: (1) the PH of the umbilical artery was ≤ 7.05; or (2) the APGAR score was ≤ 7 at 5 min after delivery and a reanimation type III or greater was required. The institutional Medical Ethics Review Board, ComitéÉtico de InvestigaciÃşn Clínica (CEIC) of the Hospital Universitario FundaciÃşn de AlcorcÃşn, approved the use of this data. Patient records/data were anonymized and de-identified prior to analysis.

**Figure 2:**
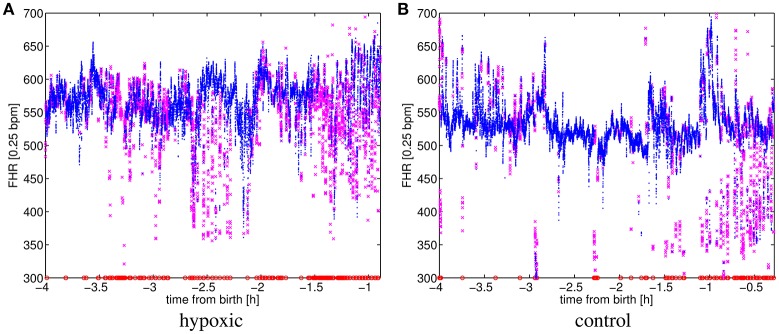
**FHR for (A)** a hypoxic and **(B)** a control patient. Signal qualities are 9.9% lost, 19.2% medium and 70.9% high for **(A)**; and 1.8% lost, 9.8% medium and 88.3% high for **(B)**. Signal qualities high, medium and lost are respectively represented by the markers: “·,” “x,” and “o.”

Records, see Figure 2 for an example, show considerable variability both at start/ending times and pauses as labor duration vary. In addition, the cardiotocograph may be disconnected at any time for a number of reasons. Also, the signal is sometimes lost as the fetus and mother move. The cardiotocograph provides three signal qualities (lost, medium and high), indicating the quality of every sample in the recording. We decided to consider the window of 4 to 1 h before birth for our analysis, even though not all patients have a signal throughout such window, e.g., nine patients began being monitored after 4 h to delivery (8 cases) or the cardiotocograph was removed before 1 h to delivery (one case). When a patient has no signal in the entire interval analyzed, she was excluded.

## 4. Results

### 4.1. Raw data analysis using NCD

In this experiment, we analyzed three types of FHR signals: (a) including only high quality signals (H); (b) also including medium quality signals (HM); (c) also including medium quality and lost (represented with a zero) signals (HML).

By using NCD, a dissimilarity matrix was created with all pairwise dissimilarities between signals, using both controls and cases. We used the software provided by NCD authors (Cilibrasi et al., 2008) to compute the NCD. The accuracy was estimated by using LOO cross-validation with a nearest neighbor classifier.

Best results are summarized in Table 1, where we see that a high quality signal and the interval from 4 to 3 h before delivery are the best for prediction accuracy (0.73). In addition, for the same time interval, we observed that the prediction using HML signal is better than using only high and medium qualities, which shows that taking into account lost signals that may occur when the fetus moves, might increase prediction accuracy. The best prediction accuracy for the complete interval (4 to 1 h prior to delivery) was obtained again considering only a high quality signal, but it did not rise above 0.66.

**Table 1:** **NCD and nearest neighbor classifier best results for raw signals**.

**Quality**	**T/C**	**Interval**	**Acc**.	**Sen**.	**Spe**.	**Compressor**	**Sym**.
H	13/13	4 ↔ 3	**0.73**	0.69	0.77	zip	Min
H	13/14	3 ↔ 2	0.63	0.57	0.69	bzip2	Min
H	15/16	2 ↔ 1	0.58	0.75	0.40	lzma	Mean
H	15/17	4 ↔ 1	0.66	0.82	0.47	zip	Min
HM	13/13	4 ↔ 3	0.58	0.62	0.54	zip	Min
HM	13/14	3 ↔ 2	0.56	0.79	0.31	bzip2	Min
HM	15/16	2 ↔ 1	0.55	1.0	0.07	lzma	Mean
HM	15/17	4 ↔ 1	0.56	0.59	0.53	lzma	Min
HML	13/13	4 ↔ 3	0.66	0.77	0.54	zip	Min
HML	13/14	3 ↔ 2	0.56	0.14	1.0	lzma	Min
HML	15/17	2 ↔ 1	0.53	0.76	0.27	lzma	Min
HML	15/17	4 ↔ 1	0.59	0.59	0.60	zip	Min

*“Quality” shows the types of signal included in the analysis (High, Medium, Low). “Interval” expresses the signal interval in hours to delivery. “T/C” shows the number of con**T**rols and **C**ases, respectively. “Acc.,” “Sen.,” and “Spe.” stand for accuracy, sensitivity and specificity, respectively; and “Sym.” shows the method used to make the dissimilarity matrix symmetric*.

*The bold value is best performance (accuracy) result*.

### 4.2. HRV and statistical moments indices using machine learnig

In this subsection, we evaluated the performance of a system using HRV and statistical moments indices as inputs of a classifier, without using NCD. This experiment aimed to evaluate whether the proposed approach using NCD showed any improvement over a classical approach.

#### 4.2.1. Time and frequency HRV indices

We computed the described HRV indices for the considered time intervals. We used all signal qualities, however in this experiment, interpolation was performed on the beats classified as artifacts, as described in Section 2.1. We then standardized (zero-mean, unit-variance) each descriptor and combined all of them into a vector. We benchmarked the following classifiers: nearest neighbor (NN), *k* nearest neighbors (*k*-NN) and SVM with linear (SVC) and radial basis function (RBF-SVC) kernels.

Table 2 shows the results of LOO cross-validation. The best performance (0.74 accuracy) using time domain HRV indices was obtained in the 3 ↔ 2 interval by a RBF-SVC classifier. The best results (0.74 accuracy) using frequency domain HRV indices were obtained in the 3 ↔ 2 interval by k-NN. The combination of the Time and Frequency indices (table not shown) gave a maximum accuracy of 0.70 in the 3 ↔ 2 interval with all classifiers but 1-NN. The best results obtained by these methods provided almost no gain over raw analysis using NCD.

**Table 2:** **HRV time and frequency indices and statistical moments accuracy for the considered time intervals**.

**Interval**	**Features**	**1-NN**	***k*****-NN**	**SVC**	**RBF-SVC**
4 ↔ 3	Time	0.69	0.69	0.35	0.5
3 ↔ 2		0.70	0.67	**0.74**	**0.74**
2 ↔ 1		0.59	0.5	0.47	0.47
4 ↔ 1		0.47	0.5	0.5	0.37
4 ↔ 3	Frequency	0.54	0.65	0.62	0.46
3 ↔ 2		0.56	**0.74**	0.67	0.70
2 ↔ 1		0.58	0.58	0.42	0.23
4 ↔ 1		0.5	0.5	0.53	0.44
4 ↔3	Moments	**0.69**	0.62	0.46	0.58
3 ↔2		0.22	0.63	0.59	0.52
2 ↔1		0.23	0.065	0.48	0.29
4 ↔1		0.5	0.44	**0.69**	0.59

*SVC and RBF-SVC stand for linear and radial basis kernel Support Vector Classifier, respectively*.

*The bold values are best performance (accuracy) result*.

#### 4.2.2. Statistical moments

In order to compute the moments on the records including high and medium signal qualities, we firstly scaled the FHR signal dividing it by the maximum value of each moment for all patients. We then calculated raw and central moments of orders *n* = {1, 2, …, 10} for each patient. Finally, the transformation $x\to \sqrt[{k}]{x}$ were applied to the moments, where *k* is the order of the moment, and standardized (zero-mean and unit-variance). The results of classifying the records with these moments are also shown in Table 2, where a 0.69 accuracy was obtained in the 4 to 3 h and 4 to 1 h before delivery intervals. Again, no real gain was obtained by this set of features over the raw analysis using NCD.

#### 4.2.3. Feature selection

Finally, we proposed a system in which all the available indices (HRV and moments) are used as feature inputs of the classifier engine. Due to sample size, and to avoid overfitting, we proposed to use feature selection, which sometimes simplifies, and even improves, the learning task. In this section, we applied forward selection (FS) to HRV (time and frequency) indices and to statistical moments. The results of applying FS to the time HRV indices were in general no better than those obtained without feature selection (see Table 3). The maximum overall accuracy was to 0.72, which was found in the interval of 2 to 1 h prior to delivery. The FS algorithm consistently selected *stdFHR* as the unique feature for classification in this case.

**Table 3:** **Accuracy results using feature selection with HRV time and frequency indices and statistical moments for the considered time intervals, without using NCD**.

**Interval**	**Features**	**1-NN**	***k*****-NN**	**SVC**	**RBF-SVC**
4 ↔ 3	Time	0.54	0.54	0.27	0.35
3 ↔ 2		0.48	0.67	0.63	0.59
2 ↔ 1		**0.72**	0.72	0.44	0.69
4 ↔ 1		0.34	0.34	0.56	0.38
4 ↔ 3	Frequency	0.62	0.62	0.077	0.65
3 ↔ 2		0.52	0.59	0.74	0.67
2 ↔ 1		0.65	0.48	0.19	0.55
4 ↔ 1		**0.75**	0.75	0.34	0.41
4 ↔ 3	Moments	**0.73**	**0.73**	0.19	0.69
3 ↔ 2		0.26	0.3	0.41	0.3
2 ↔ 1		0.42	0.39	0.42	0.42
4 ↔ 1		0.41	0.34	0.44	0.41

*The bold values are best performance (accuracy) result*.

The results for FS on the frequency HRV indices were slightly better than without feature selection. FS slightly improved the results in all time intervals (see Table 3). The best result (0.75 accuracy) was attained for the interval from 4 to 1 h to delivery. The FS algorithm consistently selected $P_{LF}/\left(P_{MF}+P_{HF}\right)$ as the single classification feature in this case.

The results of FS on the statistical moments improved the maximum accuracy obtained in Table 2 with a moderate increase (from 0.69 to 0.73). Most selected features, for the interval of 4 to 3 h to delivery, were μ_4_, μ_8_, and μ_9_.

### 4.3. HRV and statistical moments indices using NCD and a classifier

Previous results (see Sections 4.1 and 4.2) showed that using NCD on raw FHR recordings yielded to similar accuracy results as using HRV and moments plus a classifier. In this section, we assessed whether computing the NCD on sequences of HRV and moments indices, plus a classifier, could improve the final performace. This scheme corresponda with the complete system that we proposed as a contribution of this work.

#### 4.3.1. Time and frequency indices in sliding windows

Therefore, we considered the calculation of new signals from obtaining FHR indices in each time interval and evaluated the performance of obtaining the similarities of these signals for all patients with NCD and by classifying the result with nearest-neighbor. This classifier was used due to its simplicity and good results showed in the previous Section.

For each time interval, we used the NCD to analyze the time and frequency indices in 5-min sliding windows, where a window was only considered if its data did not have too many artifacts (see Section 2.1). For each parameter, a sequence was constructed for each patient by concatenating the parameter value for each sliding window of the FHR signal. An NCD matrix for each parameter was later constructed by obtaining the similarities between all pairs of patient sequences and the accuracy of a nearest neighbor classifier was estimated by leave-one-out cross-validation.

Table 4 shows the best individual accuracies of the HRV time indices in each analysis interval. The best result (0.70 accuracy, 0.86 sensitivity, and 0.54 specificity) was again in the 3 ↔ 2 interval by using the LTI index. We combined the four time indices by voting, and the best result gave an accuracy of 0.66 with sensitivity of 0.76 and specificity of 0.53 in the 4 ↔ 1 interval.

**Table 4:** **NCD and nearest neighbor classifier best results for HRV time and frequency indices and statistical moments, evaluated individually, in 5-min sliding-windows signals**.

**Interval**	**Features**	**Acc**.	**Sen**.	**Spe**.	**Feature**	**Comp**.	**Sym**.
4 ↔ 3	Time	0.62	0.69	0.54	*sdFHR*	bzip2	Min
3 ↔ 2		**0.70**	0.86	0.54	LTI	lzma	Min
2 ↔ 1		0.66	0.65	0.67	$\bar{FHR}$	bzip2	Min
4 ↔ 1		0.69	0.76	0.6	$\bar{FHR}$	bzip2	Min
4 ↔ 3	Frequency	**0.77**	0.77	0.77	$\frac{P_{LF}}{P_{MF}+P_{HF}}$	bzip2	Min
3 ↔ 2		0.59	0.64	0.54	*P*_*VLF*_	bzip2	Min
2 ↔ 1		0.69	0.65	0.73	*P*_*HF*_	bzip2	Min
4 ↔ 1		0.69	0.88	0.47	*P*_*LF*_	lzma	Mean
4 ↔ 3	Moments	**0.88**	0.92	0.85	μ_3_	lzma	Min
3 ↔ 2		0.70	0.64	0.77	μ_2_	bzip2	Min
2 ↔ 1		0.77	0.81	0.73	*M*_4_	zip	Mean
4 ↔ 1		0.81	0.82	0.80	*M*_4_	lzma	Mean

*The bold values are best performance (accuracy) result*.

Table 4 also shows the best individual accuracies of the HRV frequency indices. The best result (0.77 accuracy, 0.77 specificity, and 0.77 specificity) was in the 4 ↔ 3 interval by using $P_{LF}/\left(P_{MF}+P_{HF}\right)$. Different indices seemed to be the most informative in each interval.

#### 4.3.2. Moments in sliding windows

In this experiment, we used high and medium signal qualities and 5 min sliding windows. For each window, we computed raw and central moments of orders *n* ∈ {1, 2, …, 10}. Then, for each moment of order *n*, the results of all windows were concatenated to obtain the new signal **s**_*i,n*_ that provided a description of the patient *i*. Later, this signal was transformed as ${\bar{s}}_{i,n}=\sqrt[{n}]{s_{i,n}}/A_{n}$ where *A*_*n*_ is the maximum value of the signal $\{{\bar{s}}_{i,n}{\}}_{i\;=\;1}^{N_{T}}$. The NCD pairwise distances were then obtained for pairs $\left({\bar{s}}_{i,n},{\bar{s}}_{j,n}\right)$ and accuracies were estimated using leave-one-out cross-validation with a nearest neighbor classifier.

The results are summarized in Table 4. The best predictive interval was the 4 to 3 h to delivery. The best accuracy for individual moments gave an accuracy of 0.88, a sensitivity of 0.92 and a specificity of 0.85. In addition, we noted the good performance of the 4 to 1 h to delivery interval, which can be applied to any record of our database, with 0.81 accuracy, 0.82 sensitivity and 0.80 specificity.

## 5. Discussion and conclusions

Several indices have been proposed to analyze FHR. The most common indices are based on time domain and frequency domain methods (Signorini et al., 2003; van Laar et al., 2009). Time domain methods aim to assess the long and short term variability of the FHR, whereas frequency domain methods aim to characterize the oscillatory contributions on the FHR. Other approaches applied nonlinear techniques to characterize FHR complexity (Richman and Moorman, 2000; Baumert et al., 2004). In many cases, these indices are reduced to a single number obtained in the entire time series or to a collection of numbers obtained in 5-min window slides, which are again reduced to a few numbers such as mean or standard deviation. However, this approach has limitations in clinical practice where recordings have different time duration, interruptions and loose quality conditions.

We have proposed NCD as a similarity measure for FHR registers because it is able to exploit both linear and non-linear relations between records and is robust against the limitations of real recordings. We computed NCD on raw FHR records, as well as on feature sequences given by time and frequency indices and signal moments estimated on FHR signals. In summary, we have proposed a robust method for automated detection of perinatal hypoxia from FHR time series registered during labor.We obtained better performance from the moments than from the raw records, which shows that the compressor is not able to extract all the relations in the data and that preprocessing may help. The database we used has 32 subjects, so we use best practices to evaluate our approach using Cross-Validation for not overestimating our results.

The main advantages of using NCD for comparing FHR signals are simplicity and generality. Other commonly used information-theoretical measures, such as Approximate entropy (Pincus, 1991) or Sample entropy (Richman and Moorman, 2000), have free parameters that have to be tuned, namely, the embedding dimension and tolerance, which is a continuous parameter; but there is no parameter to tune in our approach. In addition, there is no problem with the common signal loss, which represents a problem for frequency-related methods, as they need signal interpolation, which is not always possible. The similarity can always be computed independently on how the signal loss is addressed (interpolation, concatenation, …).

It is remarkable that using sliding windows and NCD, both frequency indices and moments obtain the best accuracies in the 4 ↔ 3 h interval, whereas time indices obtain the best results in the 3 ↔ 2 h interval. Our comparisons show that the commonly used Time and Frequency indices can be complemented by the moments, which are always applicable and do not suffer from signal loss. In addition, fetus movement may provide valuable information, as we noted when analyzing raw signals (Table 1) and when we observed the performance of the $P_{LF}/\left(P_{MF}+P_{HF}\right)$ index, which depends on fetus movement (Table 4). Finally, we also performed forward selection with the sequences of indices by adding similarity matrices, and we obtained similar results, 0.88 accuracy, in the 4 ↔ 1 h interval, which compares entire records during labor. Results showed that lzma combined with statistical moment μ_3_ and 1-NN as a classifier engine was the best combinations, however, bzip2 showed a good performance in almost every combination. A detailed discussion about the selection of the compressor is done in Cebrián et al. (2005). Results of Table 4 showed that physiological conditions that lead to perinatal hypoxia are better detected at the begining of the labor. Ratio of power in different bands, as quantified by $P_{LF}/\left(P_{MF}+P_{HF}\right)$, assesses the SNA balance, therefore an alteration of this balance seemed and indicator of possible perinatal hypoxia. Moreover, since μ_3_ assess the asymmetry of the FHR distribution, it seemed that an alteration of this asymmetry could be and indicator for perinatal hypoxia. Symmetric distributions would identify fetuses with same number of times of HR samples higher and lower than the mean.

Practical implementation of this approach as a plugin to available CTG systems is straightforward. We recommend performing a careful selection and labeling of FHR records. The number of cases in the knowledge database and processing capabilities must then be balanced. For instance, the analysis of a new FHR record every minute against a large knowledge database (1,000 patients) is easily done on a normal PC using gzip as a compressor.

Decision making during labor is a difficult task for gynecologists. It should always be intended to be as less invasive as possible however, of course, ensuring fetal well-being and acting as soon as possible in the case of suspicion of fetal hypoxia. Our main contribution shows how the NCD analysis of the readily available FHR traces may help gynecologists to make the correct decisions. We reach 88% accuracy, which is a remarkable result if we take into account that we are actually identifying stressed fetuses 3 h before delivery that were not detected by the gynecologist until a later stage. This general methodology is also applicable to other time series classification problems and is both simple to understand and simple to apply.

The results obtained in this study should be confirmed on a large study, since one possible limitation is the number of subject in the database. So, further research, with more patients, should be performed to open up the application of this type of FHR analysis of the fetus condition to the industry.

The main conclusion of the work is that similarity learning, using NCD, allowed to compare sequences with different length and different quality, and it also was able to exploit the nonlinear relationship among sequences (raw FHR signals or indices). The proposed final system outperforms the classical approach using FHR indices plus machine learning, and it can be used as a framework to build robust hypoxia detectors.

## Author contributions

OB: Computing FHR indices. Manuscript writing. RS: Computing NCD, main idea. Manuscript writing. JL: Computing NCD. BG: Database and preprocessing. RG: Computing FHR indices. Manuscript writing. AC: Manuscript writing. JR: Main idea. Manuscript writing. CM: Clinical help, database, discussion.

### Conflict of interest statement

The authors declare that the research was conducted in the absence of any commercial or financial relationships that could be construed as a potential conflict of interest.

## Footnotes

^1^Data is available from the website: http://sites.google.com/site/hufahypoxia.
